# Enantioselective Gas Chromatographic Separation of Racemic *N*-alkylated Barbiturates: Application of C11-Chirasil-Dex as Chiral Stationary Phase in GC

**Published:** 2007-09-18

**Authors:** Ashraf Ghanem

**Affiliations:** Biomedicinal Chemistry Unit, Biological and Medical Research Department, King Faisal Specialist Hospital and Research Centre, (MBC-03-95), P.O. Box 3354, Riyadh 11211, Saudi Arabia.

**Keywords:** Chirasil-*β*-Dex, enantiomeric excess, gas chromatography, N-alkylated barbiturates

## Abstract

Chirasil-*β*-Dex containing an undecamethylene spacer (C11-Chirasil-Dex) was synthesized and used as chiral stationary phase (CSP) in enantioselective gas chromatography (GC). The versatility of the new stationary phase in the simultaneous enantiomeric separation of a set of *N*-alkylated barbiturates is demonstrated.

## Introduction

Barbiturates were first introduced for medical use in the early 1900s. More than 2,500 barbiturates have been synthesized, and at the height of their popularity, about 50 were marketed for human use [1]. Yet, about a dozen are in medical use. Barbiturates produce a wide spectrum of central nervous system depression ranging from mild sedation to coma. By virtue of this, they have been used as sedatives, hypnotics, anesthetics, and anticonvulsants [2]. The primary differences among many of these products are how fast they produce an effect and how long those effects last. Barbiturates are classified as ultra-short, short, intermediate, and long-acting. Their enantiomers should be of particular interest. For example, Knabe et al demonstrated that the narcotic effects of the enantiomers of *N*-alkylated barbiturates differ markedly [3, 4]. In some instances one of the enantiomers even displays convulsive properties. The enantiomers also showed different pharmacokinetic properties.

A number of synthetic methodologies have been developed to access to enantiomerically pure barbiturates [5, 6]. The ways in which efficiency and practicality of these procedures are defined is depending on a large number of factors such as scale, reagent costs, time allotted and required. Most importantly, the requirement of suitable equipments and reliable methods for the determination of the enantiomeric excesses (ee) of the resulting products. The development of accurate non-chiroptic methods for the determination of enantiomeric purity has been critical for the development of enantioselective catalysis. Thus, a prerequisite in asymmetric synthesis is a precise and reliable assessment of the enantiomeric purity of the resulting products [7, 8]. Among these methods are: polarimetric methods, gas chromatographic methods, liquid chromatographic methods and NMR spectroscopy. The modern and most sensitive methods used in the determination of enantiomeric purity of chiral drugs, allowing a detection as little as 0.1% of one enantiomer in the presence of another, are chiral GC and HPLC methods. For an efficient monitoring of the reaction progress, enantioselective gas chromatography (GC) was the method of choice for the simultaneous resolution of barbiturates enantiomers.

Although a large number of chiral stationary phases (CSPs) have been developed [9–15], the choice of an appropriate column is still difficult to achieve. Modified cyclodextrins (CDs) have been widely used as chiral stationary phases for GC separation of racemic chiral compounds. These CD derivatives are dissolved in polysiloxane phases and are used for preparing efficient capillary columns. Chirasil-*β*-Dex, a polysiloxane-anchored permethylated *β*-cyclodextrin with 3, 5 and 8 spacer have been successfully used as CSP in GC [9]. In this contribution, we report on the investigation of Chirasil-*β*-Dex with a new 11-spacer as CSP for the gas chromatographic enantiomers separation of a set of *N*-alkylated barbiturates.

## Experimental

### Preparation of Chirasil-Dex by hydrosilylation [7]

In nitrogen atmosphere, 0.56 g (appr. 0.19 mmol) dimethylpolysiloxane containing 9.3% CH_3_-Si-H groups and 0.37 g (0.23 mmol) of permethylated 11-undec-1-enyl *β*-cyclodextrin, dried in vacuo at 40 °C over P_4_O_10_ for 72 hrs, and 100 ml of dry toluene were placed into 100 ml three-necked, round-bottomed flask equipped with a nitrogen inlet and reflux condenser fitted with a mercury valve. To the refluxing reaction mixture were added a few droplets of a semi-concentrated solution of the catalyst H_2_PtCl_6_ in anhydrous THF at intervals of 150 min each. After 48 hrs reflux at 115 °C, the solvent was evaporated in vacuo with rotary evaporator yielding 1.35 g product [7].

### Materials

All *N*-alkylated barbiturates: 5-ethyl-1-methyl-5-*n*-propyl barbituric acid (**1**), 1-methyl-5-(2-propyl)-5-(n-propyl) barbituric acid (**2**), 1, 5-dimethyl-5-ethyl barbituric acid (**3**), 1,5-dimethyl-5-phenyl barbituric acid (**4**) also known as methylheptobarbital or methyleudan, 5-cyclo-hexyl-5-ethyl-1-methyl-barbituric acid (**5**), 5-(1-cyclopenten-1-yl)-5-ethyl-1-methyl barbituric acid (**6**), 1-methyl-5-phenyl-5-propyl barbituric acid (**7**), 5-(1-cyclohexen-1-yl)-1,5-dimethyl barbituric acid (**8**), 1-methyl-5-butyl-5-phenyl barbituric acid (**9**), 1-ethyl-5-methyl-5-piperidyl barbituric acid (**10**), 5-(1-cyclohexen-1-yl)-1-ethyl-5-methyl barbituric acid (**11**), 5-(1-cyclohexen-1-yl)-5-methyl-1-propyl barbituric acid (**12**) were given as a gift by Prof. Joachim Knabe, University of Saar-bruecken, Germany.

### Enantioselective gas-chromatographic analysis

A gas chromatograph (Agilent Technologies, Waldbronn, Germany) equipped with a flame ionization detector (FID) was used. The chiral stationary phase chirasil-*β*-cyclodextrin with the new 11-spacer was coated on a non-deactivated coated on 19 m × 0.25 mm fused silica capillary column (0.25 μm film thickness) according to the literature procedure [7]. The analytical conditions were: Injector temperature, 250 °C; FID temperature, 250 °C; oven temperature is varying according to the analyte itself. Hydrogen was used as the carrier gas (40 KPa column head pressure).

## Results and discussion

**Figure 1.**
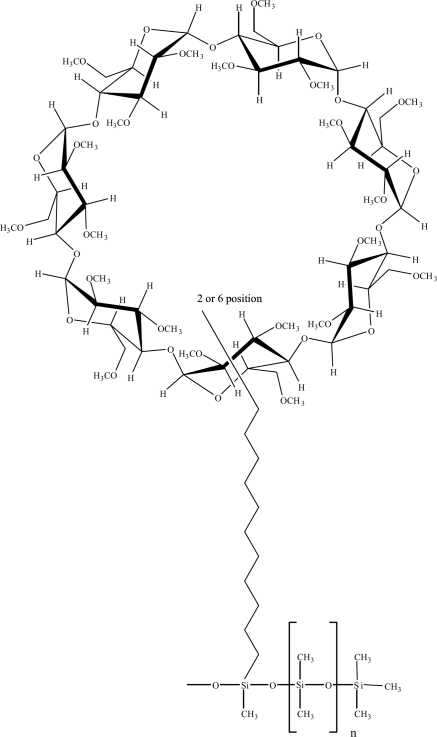
The structure of the synthesized permethylated *β*-cyclodextrin with a new C11-spacer (C11-Chirasil-Dex) bonded to a polysiloxane backbone and used as chiral stationary phase in GC.

Chirasil-*β*-Dex with a new 11-spacer (C11-Chira-sil-Dex) was synthesized and characterized by ^1^H, ^13^C-NMR and IR spectroscopy confirming the chemical link of the cyclodextrin moiety to the polysiloxane backbone [9]. Schurig and Combret et al reported that this non-regioselective Chirasil-Dex columns formulated as O-6, but now revised as a mixture of O-2-and O-6-Chirasil-Dex with a preponderance of O-2-Chirasil-Dex [16] (cf. Fig. 1).

This new chiral stationary phase was previously used to determine the enantiomeric excesses of secondary alcohols and their corresponding esters resulting from the lipase-catalyzed enantioselective transesterification in organic solvents and the intermolecular cyclopropanation of olefins catalyzed by rhodium (II) catalyst.

**Figure 2.**
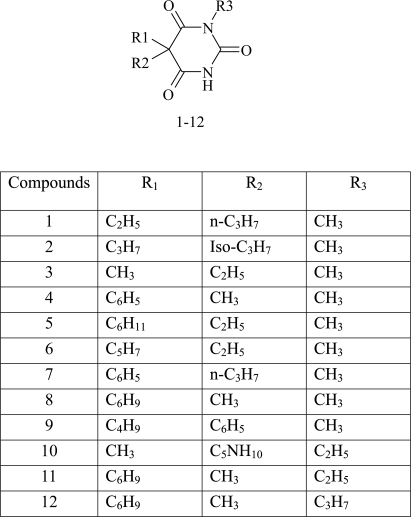
Structure of the *N*-alkylated barbiturates investigated in this study.

The enantioselective resolution of racemic barbiturates using GC and liquid chromatography using different chiral stationary phase has been previously reported [17, 18]. In our effort to discover new applications for the newly developed Chirasil *β*-dex with 11 spacer (C11-Chirasil dex), the enantioselective resolution of a set of *N*-alkylated barbiturates was investigated (cf Fig. 2).

**Figure 3.**
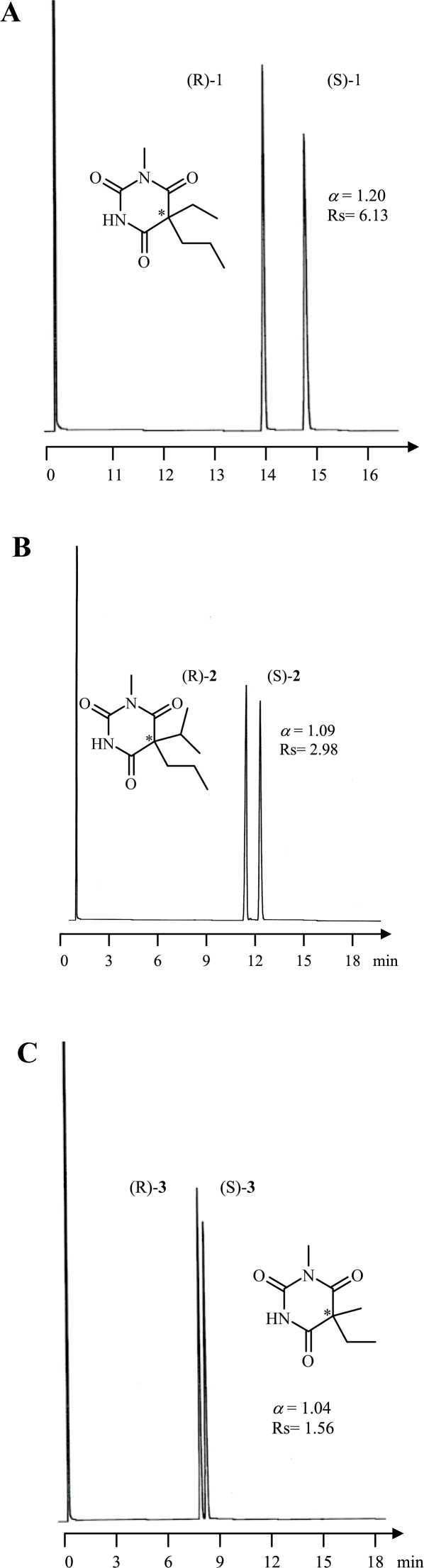
Enantioselective gas chromatographic separation of *N*-alkylated barbiturates (**1**, **2** and **3**). Oven tempereature was set at 150 °C isothermal.

**Figure 4.**
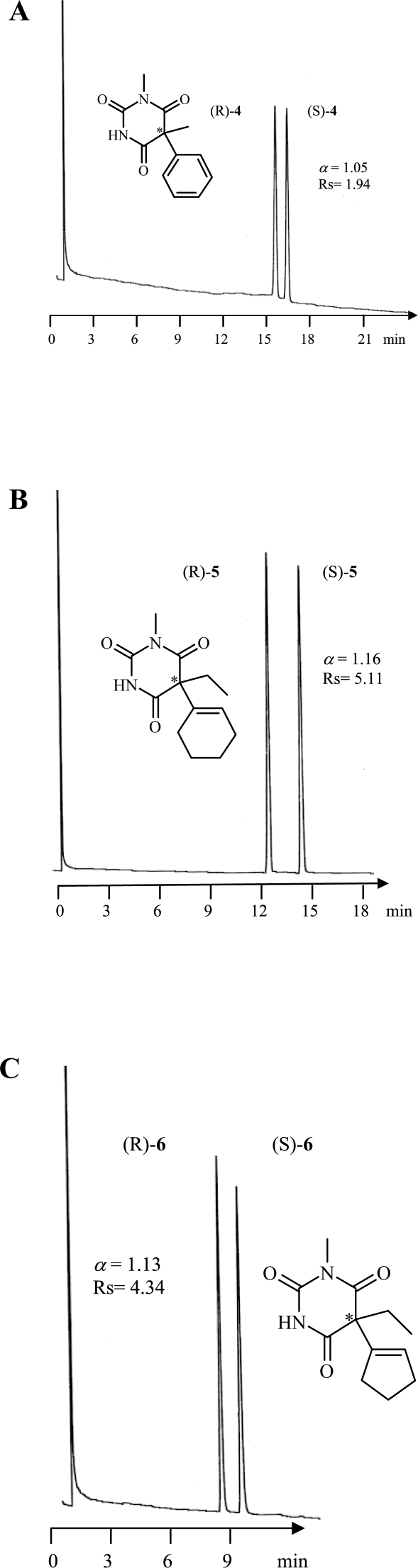
Enantioselective gas chromatographic separation of *N*-alkylated barbiturates (**4**, **5** and **6**). Oven tempereature was 180 °C isothermal.

**Figure 5.**
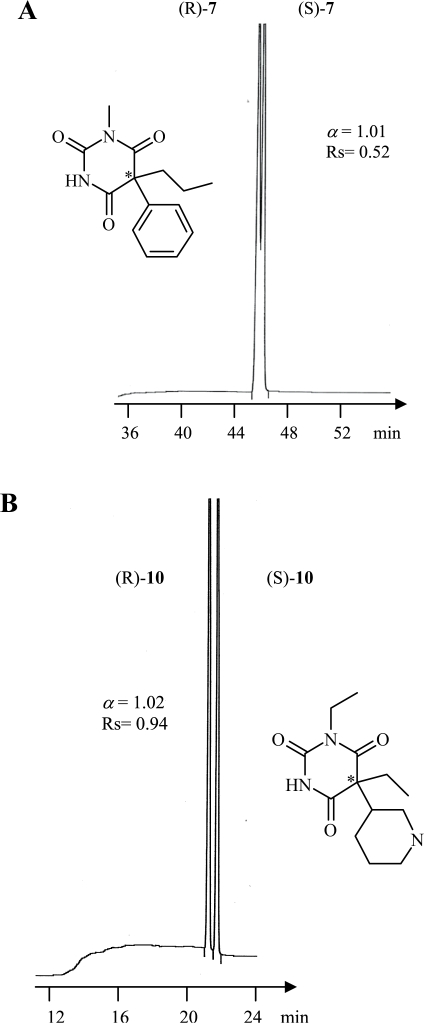
Enantioselective gas chromatographic separation of *N*-alkylated barbiturates (**7** and **10**). An oven tempereature program was applied:150 °C for 30 min followed a temperature increase of 30 °C/min to 180 °C for **7** and 150 °C for 13 min followed a temperature increase of 30 °C/min to 180 °C for **10**.

Among all racemic barbiturates investigated in this study, enantiomers of **1–6** and **10** were baseline resolved, however, **7** was partially separated (cf Fig. 3–5).

On the other hand, **8**, **9**, **11** and **12** have never been separated using different temperature program. A cyclohexen-1-yl group at C5 is in common in the structures of the unresolved racemates of **8**, **11** and **12** while a phenyl group associated with an *n*-butyl group was at C5 in the unresolved **9** and a phenyl group associated with an *n*-propyl group was at C5 in the partially separated 7. It concluded to us that the chiral recognition ability of the chiral stationary phase towards the analyzed barbiturates is depending to a large extent on the type of substituent at C5 in barbiturates structures. For example, those who process an n-alkyl group (up to n = 3) or/and a phenyl group at C5 were easily recognized by the chiral stationary phase and hence being resolved, while those having a cyclohxen-1-yl group at C5 are not recognized and unresolved. Chromatographic parameters including the separation factor (*α*) and the resolution (R_s_) are shown in Table 1. A chromatogram showing the enantioselective resolution of all investigated barbiturates in one run is demonstrated in Figure 6.

## Conclusion

The utility of polysiloxane-anchored permethylated *β*-cyclodextrins (Chirasil-*β*-dex) as CSP in GC was demonstrated in the enantioselective separation of a set of *N*-alkylated barbiturates. The developed GC method is useful for the reaction monitoring involving the synthesis of barbiturates.

## Figures and Tables

**Figrue 6.**
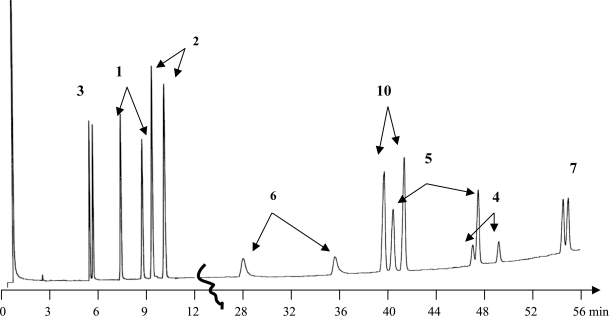
Enantioselective gas chromatographic separation of *N*-alkylated barbiturates. An oven tempereature program was applied:150 °C for 30 min followed a temperature increase of 1 °C/min to 180 °C at 50 kpa column head pressure.

**Table 1.** Oven temperature (T), retentiom time (t_R_), resolution (R_s_) and the separation factor (*α*) of the simultaneous baseline separation of racemic *N*-alkylated barbiturates.

**Compounds**	**Oven temperature^1^**	**t_R_ (***R***)**	**t_R_ (***S***)**	*R*_s_	*α*
**1**	150 °C	14.02	14.68	6.13	1.20
**2**	150 °C	11.37	12.31	2.98	1.09
**3**	150 °C	8.51	8.77	1.56	1.04
**4**	180 °C	15.58	16.28	1.94	1.05
**5**	180 °C	12.72	14.32	5.11	1.13
**6**	180 °C	9.12	10.00	4.34	1.13
**7**	150 °C/30 min., 30 °C/min to 180 °C	46.66	46.95	0.52	1.01
**8**	n.s.	n.s.	n.s.	n.s.	n.s.
**9**	n.s.	n.s.	n.s.	n.s.	n.s.
**10**	150 °C/13 min., 30 °C/min to 180 °C	22.7	23.7	1.71	1.05
**11**	n.s.	n.s.	n.s.	n.s.	n.s.
**12**	n.s.	n.s.	n.s.	n.s.	n.s.

1 The column head pressure is 40 KPa, the injector temperature is 250 °C and the FID temperature is 250 °C. n.s. (not separated)
